# The impact of 18-fluorodeoxyglucose positron emission tomography on the staging, management and outcome of anal cancer

**DOI:** 10.1038/sj.bjc.6604897

**Published:** 2009-03-03

**Authors:** E de Winton, A G Heriot, M Ng, R J Hicks, A Hogg, A Milner, T Leong, M Fay, J MacKay, E Drummond, S Y Ngan

**Affiliations:** 1 Department of Oncology, Royal United Hospital, Bath, UK; 2 Division of Surgical Oncology, Peter MacCallum Cancer Centre, Melbourne, Victoria, Australia; 3 Radiation Oncology, Peter MacCallum Cancer Centre, Melbourne, Victoria, Australia; 4 Centre for Molecular Imaging, Peter MacCallum Cancer Centre, Melbourne, Victoria, Australia; 5 Centre for Biostatistics & Clinical Trials, Peter MacCallum Cancer Centre, Melbourne, Victoria, Australia; 6 The University of Melbourne, Melbourne, Victoria, Australia

**Keywords:** anal cancer, PET CT, staging, radiotherapy

## Abstract

Accurate inguinal and pelvic nodal staging in anal cancer is important for the prognosis and planning of radiation fields. There is evidence for the role of 18-fluorodeoxyglucose positron emission tomography (FDG-PET) in the staging and management of cancer, with early reports of an increasing role in outcome prognostication in a number of tumours. We aimed to determine the effect of FDG-PET on the nodal staging, radiotherapy planning and prognostication of patients with primary anal cancer. Sixty-one consecutive patients with anal cancer who were referred to a tertiary centre between August 1997 and November 2005 were staged with conventional imaging (CIm) (including computed tomography (CT), magnetic resonance imaging, endoscopic ultrasound and chest X-ray) and by FDG-PET. The stage determined by CIm and the proposed management plan were prospectively recorded and changes in stage and management as a result of FDG-PET assessed. Patients were treated with a uniform radiotherapy technique and dose. The accuracy of changes and prognostication of FDG-PET were validated by subsequent clinical follow-up. Kaplan–Meier survival analysis was used to estimate survival for the whole cohort and by FDG-PET and CIm stage. The tumour-stage group was changed in 23% (14 out of 61) as a result of FDG-PET (15% up-staged, 8% down-staged). Fourteen percent of T1 patients (3 out of 22), 42% of T2 patients (10 out of 24) and 40% of T3–4 patients (6 out of 15) assessed using CIm, had a change in their nodal or metastatic stage following FDG-PET. Sensitivity for nodal regional disease by FDG-PET and CIm was 89% and 62%, respectively. The staging FDG-PET scan altered management intent in 3% (2 out of 61) and radiotherapy fields in 13% (8 out of 61). The estimated 5-year overall survival (OS) and progression-free survival (PFS) for the cohort were 77.3% (95% confidence interval (CI): 55.3–90.4%) and 72.2% (95% CI: 51.5–86.4%), respectively. The estimated 5-year PFS for FDG-PET and CIm staged N2-3 disease was 70% (95% CI: 42.8–87.9%) and 55.3% (95% CI: 23.3–83.4%), respectively. FDG-PET shows increased sensitivity over CIm for staging nodal disease in anal cancer and changes treatment intent or radiotherapy prescription in a significant proportion of patients.

Anal cancer is rare with an incidence around 2 in 100 000 per year. The treatment of this tumour has changed dramatically over the last 25 years (Ryan *et al*, 2000). Since the report on combined pre-operative chemo-radiotherapy (CRT) for squamous cell carcinoma (SCC) of the anus by Nigro *et al* (1974), a number of multi-centre randomised trials have confirmed its benefit (Flam *et al*, 1996; Bartelink *et al*, 1997). Complete tumour regression occurs in 68–100% of tumours, resulting in a high rate of sphincter preservation (Sato *et al*, 2005). Chemo-radiotherapy has thus become the standard of care, with surgery reserved for salvage treatment following local failure.

Accurate clinical staging in anal cancer is essential both for prognostic information and for planning of radiotherapy fields and technique. Tumour size, depth of invasion and presence of nodal metastases are key predictors of both overall and disease-free survival (Klas *et al*, 1999). Although nodal stage is related to tumour size, it also has an independent prognostic value (Frost *et al*, 1984). The accurate identification of disease extent permits precision radiotherapy delivery, ensuring the accurate coverage of disease and sparing of organs at risk.

Current standards in staging anal cancer include physical examination to determine the primary stage, with biopsy of the primary tumour and fine needle aspiration (FNA) of clinically suspicious inguinal nodes. Computed tomography (CT) is the most commonly used conventional cross-sectional imaging method to stage lymph nodes and distant metastases in the pelvis and abdomen. However, the sensitivity of CT for regional lymph nodes in anal cancer and other pelvic malignancies is approximately 50% (Weinerman *et al*, 1983; Matsukuma *et al*, 1989; Sato *et al*, 2005).

Positron emission tomography (PET) has a rapidly expanding role in oncology, with evidence for its role in the staging and management of a number of tumour types. The fact that PET imaging is functional rather than structural limits its value in local tumour staging, but numerous studies have shown an effect in the identification of regional and distant disease (Rinne *et al*, 1998; Dwamena *et al*, 1999; Gould *et al*, 2001; Hellwig *et al*, 2001; Kalff *et al*, 2002), treatment decisions (MacManus *et al*, 2001; Spaepen *et al*, 2001; Blum *et al*, 2003; Heriot *et al*, 2004), radiotherapy planning (Haustermans *et al*, 2002; MacManus and Hicks, 2003; Leong *et al*, 2006) and in the prediction of response (Di Fabio *et al*, 2005; Duong *et al*, 2006).

A role for 18-fluorodeoxyglucose positron emission tomography (FDG-PET) as a useful imaging modality for anal cancer given the limitations of CIm has been suggested. In this paper, we aim to determine the effect of FDG-PET on the nodal staging, radiotherapy planning and prognostication of patients with primary anal cancer.

## Materials and methods

### Patient population

Sixty-one consecutive staging PET scans acquired on patients referred to the Gastrointestinal Unit at the Peter MacCallum Cancer Centre (PMCC) with primary anal cancer between August 1997 and November 2005 form the basis for this study. The scans were obtained as part of routine clinical practice and identified from a prospective PET database. Eligibility criteria required PET scans to be performed before definitive treatment and within 30 days of conventional staging investigations. Referring clinicians prospectively completed a management decision proforma before PET stating their proposed management plan for the patient in the light of the current clinical and imaging information available. Referring physicians who had not completed a management plan were contacted and asked to provide a plan before performing the PET scan on that particular patient.

All patients had histologically proven localised primary SCC of anal canal or verge, were prepared to receive radical radiotherapy and had signed informed consent for entry into the PET centre database.

The collection of impact data and outcome was approved by the institutional ethics committee.

### Determination of stage

The disease was staged according to the 6th American Joint Commission on Cancer staging system (2002).

All 61 patients underwent assessment with history, physical examination and biopsy of the primary. Primary tumour size was determined by digital rectal examination (and/or examination under anaesthesia). Eight patients had endorectal ultrasound in addition to local staging. Sixteen patients with T1 tumours underwent excisional biopsy of the primary before PET.

Conventional imaging (CIm) for regional and distant metastasis included imaging of the pelvis, abdomen and chest. Fifty-nine (97%) patients underwent cross-sectional imaging of the pelvis (41 CT alone, 17 CT and magnetic resonance imaging (MRI), and 1 MRI alone). The two patients in whom no pelvic imaging was performed both had T1 tumours and were stage 1 on PET. Owing to the prevalence of reactive lymphadenopathy in the inguinal region, we considered lymph nodes up to 15 mm to be within normal limits on CT criteria. The inguinal nodes considered as involved on clinical examination were confirmed either with fine needle aspiration cytology (FNAC) or with excisional biopsy. Sixty-three percent of the inguinal nodes reported as involved on CT were confirmed histologically.

Fifty-eight (95%) patients underwent a CT of the abdomen and 46 (75%) underwent chest imaging (35 CT, 11 CXR) to stage for distant disease. Computed Tomography staging for distant disease is now considered standard of care; this was not the case in the initial study period. However, given the very low incidence of metastatic disease at presentation in anal cancer, this is unlikely to influence the results. The three patients who did not have CIm for distant disease in the abdomen were staged as N0.

All 61 patients underwent a staging PET scan, of whom 36 had PET and 25 had PET CT.

### FDG-PET imaging and interpretation

All patients fasted for 6 h before this study but were encouraged to drink water. Patients were catheterised when possible and were given 10–20 mg furosemide 30 min before imaging to minimise the confounding effects of changing bladder activity on pelvic assessment. Patients also received bowel preparation before the procedure.

PET scans were acquired on a GE QUEST 300-H scanner (UGM Medical Systems, Inc., Philadelphia, PA, USA) and PET CT scans on a dedicated PET-CT scanner (Discovery; GE Healthcare, Chalfont, St Giles, UK) at least 1 h after intravenous injection of 300–400 MBq of 18F-FDG. The measured resolutions of PET and PET-CT were comparable, and although attenuation correction and anatomical correlation on PET-CT provide greater confidence in the detection and localisation of disease, the data are reasonable to combine as technology continues to improve and this represents a likely worst case scenario with respect to the benefits of PET/CT over conventional evaluation.

Transmission and emission scans were obtained from the lower neck to the upper thigh. Patients were scanned with their arms raised, if they could tolerate it. Emission data were processed using iterative reconstruction (Fulham *et al*, 1997) (ordered-subset expectation maximisation method) with attenuation correction (Benard *et al*, 1999). Robust co-registration of non-contemporaneous PET and CT data used rigid, mutual information matching. As the pelvic lymph nodes are relatively immobile and in a relatively fixed geometry in reference to bone anatomy, we believe that this approach is valid.

Image datasets were reported from the screen, both with and without attenuation correction, using an interactive display programme that allows multiple orthogonal images to be shown simultaneously. Rotating count-rendered images were also reviewed to aid clarification of the relationship between the physiological radiotracer accumulation and tumour in the rectum.

All PET and PET-CT studies were reported at the time of the scan by experienced PET specialists. The PET stage was determined by incorporating the PET or PET-CT findings with all other staging information available at the time of clinical reporting. This was abstracted from the clinical report issued thereafter. As per usual clinical practice, the scans were read blinded to the results of subsequent tests and the final outcome of the patient. Significant structural imaging abnormalities did not influence the PET or PET-CT result if there was no associated FDG PET or PET-CT metabolic abnormality. For chest, abdominal or pelvic activity, the focal uptake of 18F-FDG had to be greater than the mediastinal uptake, and needed to correspond to an anatomical structure or abnormality identified on CT; for example, a lymph node of normal or abnormal size. An activity less than the mediastinal activity was defined as abnormal only if there was a definite structural abnormality of <1 cm in size. This assessment is justified because of the known partial volume effect of PET-CT caused by its limited resolution below 1 cm.

### Assessment of PET impact

The PET request form incorporated a management decision proforma that required the referring physician to state tumour histology, results of structural imaging investigations, and the TNM and group stage thereby obtained, as well as the proposed management plan and intent based on CIm if PET were unavailable. The post-PET plan and intent were determined from the medical record or through direct contact with the referring clinician. The PET scan result was subsequently compared with the CIm result by stage group, nodal stage and the actual management implemented. Changes in the radiation field or technique were assessed from a review of the radiotherapy treatment sheets and planning data.

The effect on management was considered high when the treatment intent was changed from palliative to curative or visa versa, medium when the method of treatment delivery was changed, such as a change in radiation field size or technique, and low when the PET results did not indicate a need for management change. PET was considered to have had no effect when the management plan was not changed despite being inconsistent with the post-PET stage. This system of assessing PET impact by our institution has been validated and published for other tumours, including lung cancers (MacManus *et al*, 2001), non-Hodgkin's lymphomas (Blum *et al*, 2003) and oesophageal cancers(Duong *et al*, 2006).

### Chemo-radiation

All patients appropriate for radical treatment were managed with definitive radiation combined with 5-FU and mitomycin-C according to the hospital protocol or within trial. Radiation was delivered by an external beam using 6 or 18 MV photons to a total dose of 54 Gray (Gy) in 1.8 Gy daily fractions, five fractions per week using a three-phase technique. Phase 1 consisted of anterior/posterior parallel opposed fields, with the clinical target volume (CTV) including the primary tumour, perirectal, inguinal and iliac lymph nodes to a dose of 36 Gy, and with the upper field border 4 cm superior to gross disease (primary or nodal). Phase 2 used a three-field technique to the posterior pelvis with the same upper field borders, with CTV encompassing the gross disease, perirectal and iliac nodes to a dose of 45 Gy. Phase 3 treated the primary disease to a total dose of 54 Gy, using a reduced field size (2 cm on gross disease). The involved inguinal nodes were boosted to 54 Gy using electron fields. Patients with only stage 1 disease received the same total doses to primary tumour, perirectal and iliac nodes using a two-phase posterior pelvis technique.

Data from PET were used to assist in radiotherapy treatment planning, but image co-registration was not used. The standard concurrent chemotherapy was 5-FU 1 g m^–2^ for 4 days in week 1 and 5, and mitomycin C 10 mg m^–2^ intravenously on the first day of treatment only. Some patients received concurrent chemotherapy within a clinical trial using continuous infusion 5-FU 300 mg m^–2^ for 96 h every week throughout the course of radiotherapy, and mitomycin C 10 mg m^–2^ intravenously on the first day of treatment only.

### Follow-up and validation of results

Patients were followed up by review of their case notes and through contact with their referring physician to determine clinical outcomes. When appropriate, details of the date and cause of death were obtained. The site and date of any progression were recorded.

Confirmation of the presence, absence or equivocal status of disease at each site was determined by predefined protocol criteria for both CIm and PET. Methods of validation of accuracy were defined in the protocol and included pathology, therapeutic response, imaging, clinical follow-up and concordance between CIm and PET. Concordance was used if CIm and PET were negative at a nodal site within the radiation treatment field. Sites were considered not assessable by CIm if not imaged as part of follow-up (with the exception of the primary and inguinal nodes that were considered assessable by clinical examination).

### Statistical methods

The proportion of patients having a change in stage group after the staging PET scan was calculated together with the 95% confidence interval (CI), calculated using the exact methods for binomial distribution.

Overall survival (OS) was measured from the date of the staging PET scan to the date of death from any cause. Progression-free survival (PFS) was measured from the date of the staging PET scan to the date of first progression at any site or death from any cause. All patients were followed up to a study close-out (study censor) date of 17 August 2006. The survival times of those patients not experiencing the relevant event (death and/or progression) by the close-out date were censored on that date.

The Kaplan–Meier product limit method was used to estimate OS and PFS, and 95% CI for the proportion of patients surviving at particular times was estimated using the logit transformation. The Mantel–Cox log-rank test was used to compare PFS according to stage group and nodal stage assessed using CIm and PET. The reverse Kaplan–Meier method was used to estimate the potential follow-up time. All statistical analyses were conducted using StatXact (Cambridge, MA, USA: Cytel Software Corporation; 2003) and S-Plus (Seattle, WA, USA: Mathsoft; 1999) statistical software.

## Results

The median age of the 61 patients with primary anal cancer who underwent ^18^F-FDG PET was 57 years (range 27–88 years), and 56% (34) were female and 44% (27) were male.

### Staging

Excisional biopsy had been carried out before PET in 16 patients. The primary tumour was identified by PET in 100% of the remaining cases (45 out of 45). However, as discussed earlier, PET was not used to formally determine the T stage. Stage by CIm and with the addition of PET are recorded in Table 1. Stage group was changed by PET in 23% (14 out of 61) of patients (95% CI: 13–35%). Fifteen (9 out of 61) percent of patients were up-staged and 8% (5 out of 61) of patients were down-staged. In 77% (47 out of 61), the stage group was unchanged (Table 2).

Changes in the nodal stage were greater for tumours with a more advanced T stage. Only 14% (3 out of 22) of patients with T1 tumours had a change in nodal stage after PET, whereas 42% (10 out of 24) and 38% (6 out of 16) of patients with T2 and T3/4 tumours, respectively, had a change in nodal (regional or metastatic) stage after PET.

The accuracy of the post-PET nodal stage could be validated in the majority of cases (98, 94, 90 and 89% for intra-abdominal, inguinal, perirectal and iliac sites of disease, respectively). PET results of the regional nodal sites not validated were not assessable because of discordance with CIm results (or, in two cases, no CIm of the pelvis) at sites encompassed by radiation fields. In only one case was the PET intra-abdominal nodal result unable to be validated as a true negative, as the patient died within 12 months of follow-up of unrelated causes. In all cases where PET and CIm staging for metastatic disease differed, PET was subsequently validated as correct.

When compared with CIm alone, the addition of PET gave superior sensitivity for staging in anal cancer. The overall sensitivity for detection of regional nodal metastases was 89 *vs* 62% for PET *vs* CIm. Sensitivity for PET *vs* CIm for the detection of perirectal, inguinal, iliac and intra-abdominal nodes was 67 *vs* 50%, 100 *vs* 85%, 100 *vs* 50% and 100 *vs* 0%, respectively. In four patients intra-abdominal CIm staging could not be validated, three had no conventional abdominal imaging as part of staging and one died within 12 months of follow-up. Twenty-five percent (15 out of 61) of patients did not have chest imaging as part of staging and, therefore, this was not included in the analysis of accuracy.

### Management

Ninety-seven percent (59 out of 61) of patients were treated with radical intent, of which 97% (57 out of 59) received definitive CRT. One patient with stage 1 disease was treated with excision biopsy alone (patient decision) and one patient with systemic lupus received a lower neoadjuvant dose of CRT followed by definitive surgery. Of the 3% (2 out of 61) of patients treated with palliative intent, one patient with advanced loco-regional disease received palliative dose RT alone because of medical co-morbidity and one patient with metastatic nodal disease received high-dose palliative CRT.

**Figure 1:**
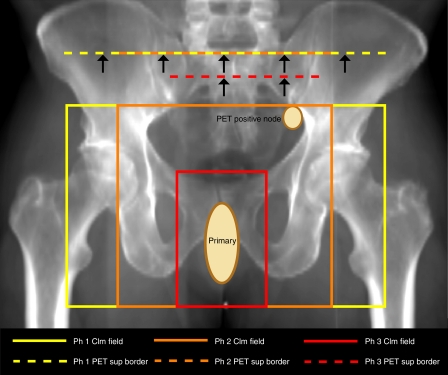
Radiograph with schematic representation of primary and PET-detected node and showing three-phase pelvic RT fields for stage by CIm (T3N0M0) and change in superior borders by PET stage (T3N2M0).

**Figure 2:**
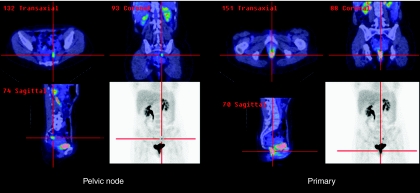
PET scan showing primary; the left iliac node metastasis was not identified on CIm and changed RT fields.

PET changed management in 16% (10 out of 61) of cases. The addition of PET to CIm staging had a high impact, changing treatment intent in 3% (2 out of 61) of patients. PET staging had a medium impact, changing radiotherapy fields or technique to cover or exclude nodal disease in 13% (8 out of 61) of patients (Figures 1 and 2). The results of PET were ignored (that is, no impact) in management in 8% (5 out of 61) of patients. In these patients, PET stage changes would potentially have altered management intent or treatment fields, but were ignored. Table 3 shows PET changes in nodal stage by T stage, the effect on management and details of the resulting changes in radiation fields. For the remaining 77% (47 out of 61) of patients, PET did not affect on the earlier planned management because the PET results were concordant with those of CIm.

### Prognostication

The median potential follow-up from the date of the PET scan to the close-out date was 2.6 years (95% CI: 2.1–3.1 years). All 61 patients were included in the survival analysis. By the close-out date of 17 August 2006, eight had died and a further four had progressed with disease.

**Figure 3:**
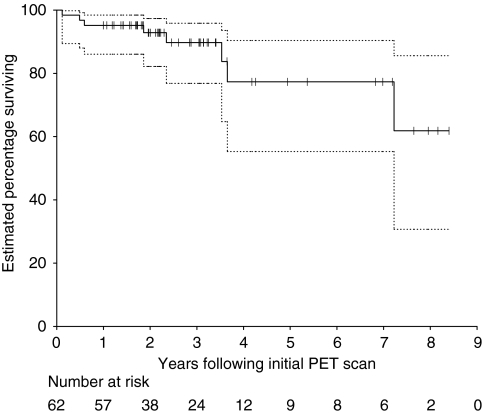
Kaplan–Meier curves of overall survival for all patients. Ninety-five percent confidence intervals are shown by the dotted lines. Patients with censored times are shown by tick marks.

**Figure 4:**
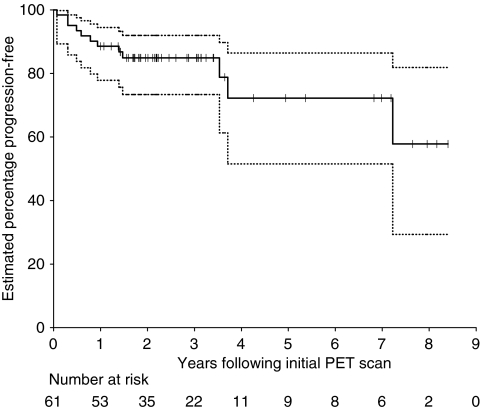
Kaplan–Meier curves of progression-free survival for all patients. Ninety-five percent confidence intervals are shown by the dotted lines. Patients with censored times are shown by tick marks.

Estimated 5-year OS and PFS were 77.3% (95% confidence interval : 55.3–90.4%) and 72.2% (95% CI: 51.5–86.4%), respectively (Figures 3 and 4).

**Figure 5:**
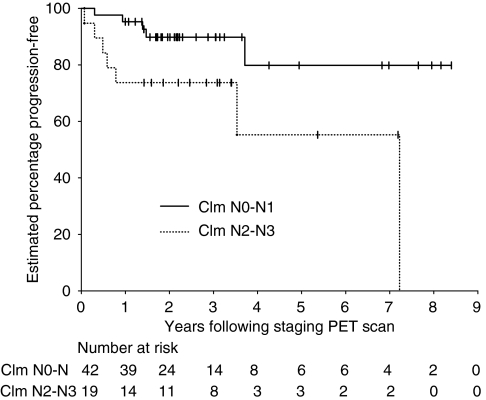
Kaplan–Meier curves of progression-free survival by N stage by CIm (*P*=0.016, log-rank test).

**Figure 6:**
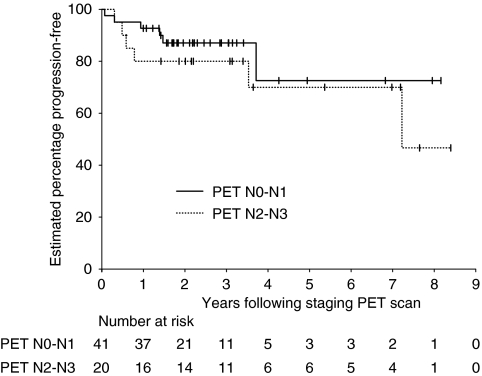
Kaplan–Meier curves of progression-free survival by N stage by PET (*P*=0.373, log-rank test).

Progression-free survival was analysed by nodal stage, as staged by CIm (Figure 5) and PET (Figure 6). Owing to small numbers, the nodal stage was grouped as N0–N1 and N2–N3. Of the 61 patients, 42 were CIm N stage 0–1 (five progressed or died) and 19 were CIm N stage 2–3 (seven progressed or died). The 3-year PFS rate was 89.8% (95% CI 75.7–96.1%) for N stage 0–1 and 73.7% (95% CI 50.2–88.6%) for N stage 2–3.

Of the 61 patients, 41 were PET N stage 0–1 (six progressed or died) and 20 were PET N stage 2–3 (six progressed or died). The 3-year PFS rate was 87.1% (95% CI 72.3–94.5%) for N stage 0–1 and 80.0% (95% CI 57.2–92.3%) for N stage 2–3 (Figure 6).

In patients with N2–3 disease, the estimated 5–year PFS for FDG-PET and CIm was 70% (95% CI: 42.8–87.9%) and 55.3% (95% CI: 23.3–83.4%), respectively.

## Discussion

As shown with other tumour types (Dwamena *et al*, 1999; Gould *et al*, 2001; Hellwig *et al*, 2001; MacManus *et al*, 2001), this study too showed that the addition of PET to the staging of patients with SCC of the anus changes stage in a substantial proportion. Overall, 23% of cases had a change in stage group and 40% of patients with T2 or larger tumours had a change in nodal stage as a result of PET. The accuracy of these nodal stage changes by PET could be validated in 93% of patients.

There is currently limited published data regarding the clinical impact of PET in addition to conventional staging for anal cancer. Trautmann *et al* reported 21 patients with anal cancer, a quarter of whom were found to have involved pelvic nodes on PET not seen on CT, although the group stage was changed only in about 10%. The lower rate of stage change in this study may well have been because of the lack of attenuation correction of PET scans, which would limit sensitivity in detecting deep pelvic lymph node disease. Cotter *et al* (2006), reported 41 patients staged with CT and FDG-PET/CT and found PET-upstaged inguinal nodes in 17% of cases in which both CT and physical examination were negative. In our cohort of 61 patients, the addition of PET showed superior sensitivity for regional node staging compared with CIm alone (89 *vs* 62%, respectively). The sensitivity of PET *vs* CIm for inguinal node metastasis was 100 *vs* 85, with 63% of patients having histological confirmation.

The limitations of PET in identifying peri-rectal nodal disease (N1) are recognised. In our cohort, the sensitivity of PET for peri-rectal nodes was 67%. The spatial resolution of PET (10 mm) and the spillover of activity from the primary tumour limit the detection of these lymph nodes adjacent to the primary. The true accuracy of peri-rectal node detection by PET may not be relevant, as perirectal nodes, involved or not, will routinely be included in the high-dose radiation treatment volume.

It is well recognised that the risk of nodal involvement increases in tumours with size >2 cm (⩾ stage T2). In tumours with size >2 cm, nodal involvement rose to 45–50%, justifying the common practice of prophylactic inguinal and iliac nodal irradiation in these patients. Some recent literature reports excellent disease control for involved field RT without prophylactic nodal irradiation in T1 tumours in 21 patients (Hatfield *et al*, 2008) or suggests reducing the target volume to the anal canal and/or the lower perirectal nodes (Ortholan *et al*, 2005). However, we found that even in T1 tumours of the anal canal there was a 10–15% risk of nodal involvement, which is not clinically insignificant and may caution against treatment to the primary alone in these patients. In contrast, none of the T1 tumours of the anal verge had nodal involvement on either CIm or PET.

Without surgical nodal staging, it is not possible to determine the specificity of PET (true negative rate). As all nodal areas were treated prophylactically, follow-up and imaging could not be used as surrogates and all discordant results could not be verified by biopsy, no attempt was made to estimate the specificity. However, studies in other pelvic malignancies have shown PET to be highly specific for nodal spread, with specificities of 95% for inguinal nodes in vulval cancer (Cohn *et al*, 2002) and over 90% for pelvic nodes in cervical cancer (Reinhardt *et al*, 2001). Low FDG uptake in inguinal nodes is sufficient, in our experience, to exclude malignancy, but if critical to patient treatment decisions, excisional biopsy should be carried out. The poor outcome of the two patients with para-aortic nodes found by PET but not CT also adds weight to the positive predictive value of PET and is consistent with a recent study in which the specificity of PET for para-aortic nodes was again shown to be 95% (Rose *et al*, 1999), as well as highlights its strength as a whole-body screening modality in excluding or detecting systemic metastases.

PET is relatively insensitive for involved nodes with size <8 mm, because of partial volume effects; however, these are also below the size limits for a positive node on both CT and MRI. Thus, false-negative results on PET are also false negative or at best equivocal on CT and MRI. The real advantage of PET lies in the high positive predictive value of significantly increased uptake in non-enlarged nodes.

Although new MRI sequences and the use of lymphatic contrast agents (for example, USPIO) may increase the sensitivity for nodal disease, these remain under investigation and have not yet entered routine clinical practice. However, they may well provide complementary anatomical staging information to PET in the future.

More recently, the question of using sentinel lymph node biopsy (SLNB) to determine those patients in whom prophylactic inguinal node irradiation can be safely omitted has been raised. A recent review of five studies of SLNB in anal cancer (Damin *et al*, 2006) concluded this technique as safe, effective and potentially important in guiding management. These studies are limited by their small numbers and their inability to determine the sensitivity of the technique. However, in contrast to breast cancer and melanoma, for which SLNB is now standard of care, surgical management of involved nodes is not standard in anal cancer, and the difficulty in obtaining adequate experience to validate results may ultimately limit its usefulness in this disease.

Treatment intent was only changed in 3% of patients, as anal cancer is predominantly a locoregional disease at presentation. However, radiation fields were altered as a result of PET in 13%. For the larger T3/4 tumours, management was changed in 100% of cases in which PET altered nodal stage. The reliance on follow-up and survival data to validate PET findings and appropriateness of management changes has limitations. Retrospective assessment of outcomes can be flawed and a long timescale between diagnostic tests and clinical outcomes makes this more difficult. Despite this, our findings still strongly suggest that management changes influenced by PET findings were usually appropriate.

The benefit from modifying radiation treatment volumes to incorporate areas found to be abnormal by PET, but not by CT, cannot be independently assessed by this study. However, intuitively, failure to treat active sites of disease ought to be detrimental. In terms of allowing individualisation of treatment, PET is an attractive adjunct.

The dose of radiation concurrent with chemotherapy required to treat anal cancer is open to debate. Using our centre's technique, inguinal nodes were routinely treated to at least 36 Gy and lower iliac nodes treated to 45 Gy prophylactically for all but the lowest risk patients (stage I). All known gross diseases were treated to 54 Gy. This approach seems to be justified by the excellent results from our cohort, however, lower doses may be adequate to reduce the risk of toxicity. There is evidence to suggest that doses as low as 30 Gy in combination with chemotherapy are sufficient for microscopic diseases (Hu *et al*, 1999), and that some anal cancer techniques, such as that used in the UK ACT II study, treat nodal areas only 30.6 Gy prophylactically. A recent publication suggests that these doses may even be sufficient to treat non-bulky primary disease (Hatfield *et al*, 2008). The effect of PET changes in treatment fields and the influence of these on outcome are, therefore, potentially greater in centres where prophylactic doses to nodal areas are lower because of differing doses and techniques.

Nodal stage as assessed by CIm was significantly associated with PFS in our cohort. However, PFS using PET nodal stage was not statistically significantly different between N0–1 and N2-3. This loss of stratification by PET nodal stage may reflect better staging and, therefore, more accurate treatment of nodal sites, although this is impossible to prove. Alternatively, it may just be because of the small numbers and short follow-up with few events. Improved 5-year PFS for FDG-PET-staged N2–3 disease compared with CIm-staged N2–3 disease could be consistent with more accurate nodal staging and appropriate radiotherapy delivery.

This study does not provide information on the independent role of PET compared with conventional staging, because the PET results were interpreted in conjunction with other clinical information. However, it does assess the incremental diagnostic information provided by PET and its effect on patient management in routine clinical practice. Accordingly, the study design reflects the situation in which PET might be clinically applied based on selection of patients with locally advanced disease and an intermediate to high likelihood of occult nodal or metastatic disease. Although PET is a valuable tool, it is expensive and, in most centres, remains a limited resource. Therefore, the potential effect must be balanced with the financial cost. This was not a formal health economics study and hence the specific economic costs have not been addressed. However, the ability to refine and individualise treatment through more accurate pre-treatment staging has the potential to improve outcome and reduce recurrence, which would result in economic savings.

Stratification of patients according to local tumour stage, with PET used in patients with tumours that are at least T2 or when regional nodal spread is suspected on structural imaging, may yield an acceptable level of stage and management changes to justify the inclusion of PET on a financial basis rather than purely a clinical one. These questions should be addressed by further study.

In conclusion, this study is the largest published series to date on the use of PET with SCC of the anus and showed a change in nodal stage and subsequent radiation fields with the addition of PET to conventional staging in a significant proportion of patients. The effect of PET is greater in primary tumours of size >2 cm as the risk of nodal involvement is low for smaller lesions. Longer follow-up and larger patient numbers will be needed to evaluate the role of PET in assessing anal cancer prognosis. With treatment strategies in anal cancer moving towards lower radiation doses and image-guided radiotherapy treatment planning, the addition of PET should be considered in any staging algorithm for all but the smallest anal tumours.

## Figures and Tables

**Table 1:** Stage group and TNM stage by CIm and PET

**Stage group**	**TNM stage**	**CIm Stage**		**PET Stage**	
		**Stage group**	**TNM**	**Stage group**	**TNM**
		**No. (%)**	**No. (%)**	**No. (%)**	**No. (%)**
I	T1N0M0	20 (33)	20 (33)	19 (31)	19 (31)
II	T2N0M0	17 (28)	14 (23)	16 (26)	14 (23)
	T3N0M0		3 (5)		2 (3)
IIIA	T1N1M0	6 (10)	0 (0)	5 (8)	0 (0)
	T2N1M0		2 (3)		1 (2)
	T3N1M0				
	T4N0M0		0 (0)		1 (2)
			4 (7)		3 (5)
IIIB	T4N1M0	17 (28)	1 (2)	19 (31)	1 (2)
	AnyTN2M0		7 (11)		12 (20)
	AnyTN3M0		9 (15)		6 (10)
IV^a^	AnyTAnyNM1	1 (2)	1 (2)	2 (3)	2 (3)
Total		61	61	61	61

Abbreviations: CIm=conventional imaging; PET=positron emission tomography; RT=Radiotherapy; TNM=Tumour, Nodes, Metastases.

a M1 disease identified was distant nodal metastases. No visceral metastases were identified.

**Table 2:** Comparison of stage group for the 61 patients by CIm *vs* PET

	**Number of patients by stage group using CIm**					
		**I**	**II**	**IIIA**	**IIIB**	**IV**
Number of patients by stage group using PET	I	18	0	0	1	0
	II	0	13	2	1	0
	IIIA	0	1	3	0	1
	IIIB	2	3	1	13	0
	IV	0	0	0	2	0

Abbreviations: CIm=conventional imaging; PET=positron emission tomography.

**Table 3:** PET changes in nodal stage by T stage and impact of changes on management

**CIm nodal stage**	**PET nodal stage**	**Impact**	**RT field changes due to PET**
*Primary tumour stage*			
*T1*			
N0	N3 (ili)	None	Radiation fields planned as for stage 1 disease. (i.e. 2 phase posterior pelvis with no prophylactic inguinal node RT)
N0	N2 (R ili)	None	
N3 (bil ili)	N0	Medium	Phase 3 boost to primary only
			
*T2*			
N0	N2 (ili)	None	Phase 1 and 2 fields upper border not changed. Phase 3 boost to primary only
N3 (ing+ili)	N3M1 (PAN)	None	Patient treated with radical intent (PAN not treated). Subsequently progressed in PAN
N1	N0	None	Peri-rectal nodes remote from primary. No phase 3 boost field size reduction from phase 2
N0	N1	Low	Proximity of peri-rectal nodes to primary meant included in phase 3 boost to primary without requiring change in field size
N1	N0	Low	
N2 (R ing)	N3 (ing+p-r)	Low	
N3 (ing+p-r)	N2 (R ing)	Low	
N0	N3 (R ili+p-r)	Medium	Superior border increased phase 1 and 2 fields. R ili node covered in phase 3 boost
N2 (R ing)	N0	Medium	No phase 1 field change or phase 2/3 electron boost to R groin
N3 (bil ing)	N2 (R ing)	Medium	No phase 1 field change or phase 2/3 electron boost to L groin
			
*T3/4*			
N0^a^	N2 (L ili)	Medium	Superior border phase 1 and 2 fields increased to cover iliac nodes. L ili covered in phase 3 boost
N3 (bil ing)	N2 (R ing)	Medium	No phase 1 field change or phase 2/3 electron boost to L groin
N0	N2 (L ing)	Medium	Phase 1 field change and phase 2/3 electron boost L groin
N3 (L ing p-r)	N3 (bil ing/ili)	Medium	Phase 1 and 2 field changes. Phase 2/3 electron boost to bilateral groins
N3M1 (ing +PAN)	N1M0	High	Intent changed from palliative to radical
N3M0 (p-r,ing +ili)	N3M1 (PAN)	High	Intent and dose changed from radical to palliative

Abbreviations: bil, bilateral; CIm=conventional imaging; Ili, iliac; ing, inguinal; L=left; PET=positron emission tomography; p-r, peri-rectal; PAN, para-aortic nodes; R=right; RT=Radiotherapy; TNM=Tumour, Nodes, Metastases.

a Changes to radiotherapy fields and PET scan for this patient are shown in Figures 1 and 2.
